# Temperature and Sugar Feeding Effects on the Activity of a Laboratory Strain of *Aedes aegypti*

**DOI:** 10.3390/insects10100347

**Published:** 2019-10-16

**Authors:** Irvin Forde Upshur, Elizabeth Annadel Bose, Cameron Hart, Chloé Lahondère

**Affiliations:** Department of Biochemistry, Virginia Polytechnic Institute and State University, Blacksburg, VA 24061, USA; uforde96@vt.edu (I.F.U.); eliza97@vt.edu (E.A.B.); cameron8@vt.edu (C.H.)

**Keywords:** actometer, abiotic factors, total carbohydrates, disease vector insects, invasive species, mosquito

## Abstract

*Aedes aegypti* is an invasive mosquito species that is expected to expand its global distribution through climate change. As poikilotherms, mosquitoes are greatly affected by the temperature of the environment which can impact host-seeking, blood-feeding, and flight activity as well as survival and ability to transmit pathogens. However, an important aspect of mosquito biology on which the effect of temperature has not been investigated is water and sugar-feeding and how access to a sugar source might affect the insect’s activity and survival under different thermal conditions. To close this knowledge gap, we relied on actometer experiments to study the activity of both female and male *Ae. aegypti* at 20 °C, 25 °C, and 30 °C, providing either water or 10% sucrose to the insects. We then measured the total carbohydrate contents of alive mosquitoes using the anthrone protocol. Survival was assessed and compared between all groups. Results from this study will inform on the thermal biology of *Ae. aegypti* mosquitoes and how access to sugar affects their activity.

## 1. Introduction

The average global surface temperature is projected to increase throughout the 21st century [1]. This might lead to longer infection seasons and expansion of multiple vectors’ geographic distributions, resulting in an increase of vector-borne disease risk. Regions at risk include much of Africa and Central and South America [2], as well as North America [3]. A disease vector insect of particular concern is *Aedes aegypti* (Linnaeus in Hasselquist, 1762), which can transmit dengue, chikungunya, Zika, and yellow fever [4]. Chikungunya and dengue are of growing global public health concern as a consequence of their recent geographical spread [5]. *Ae. aegypti* distribution is currently the widest ever recorded [6]. It has expanded widely Northward in the United States since 1995 [7] and is projected to expand substantially around the world [8]. Moreover, the number of people at risk is expected to significantly increase, with Australian, European, and North American populations expected to have the largest proportional increase in exposure (63–80% by 2061–2080) [9].

As mosquitoes are poikilotherms and thus greatly affected by temperature, the changes in distribution of *Ae. aegypti* due to climate change [6,9,10] requires further investigation about the effects of temperature on *Ae. aegypti* physiology and behavior. The effects of temperature on some aspects of *Ae. aegypti* host-seeking, blood-feeding, and flight activity have been characterized (as reviewed in [11], as well as its effects on reproductive activity and survival [12]). However, the effect of temperature on water intake and sugar-feeding, a behavior to which both male and female *Ae. aegypti* have an evolutionary commitment [13], has not been previously investigated.

Despite the fact that water does not provide nutrients, water uptake is a common behavior in both female and male mosquitoes allowing them to avoid dehydration and death, in particular under dry conditions and in the absence of other water sources such as nectar or blood [14,15]. It is essential to maintain their water balance and it has been shown to be critical for minimizing dehydration in diapausing mosquitoes [14]. Water is imbibed in small volume and passed into the midgut while nectar is usually stored in the dorsal diverticula and is transferred to the crop as needed [16,17,18,19].

Sugar-feeding is an important and physiologically relevant behavior for both male and female mosquitoes as their fitness depends on having a diet optimal in quality and quantity [20]. Energy from sugar-feeding is essential for mosquito flight energy [13,21], flight duration and distance [22], host-seeking, blood-feeding, maintenance, and reproductive success [13,21]. Mosquitoes obtain sugar by feeding on a wide range of carbohydrate sources including floral and extrafloral nectar, rotting or decaying fruit, tree sap, and honeydew [13,23,24,25,26]. Additionally, mosquitoes have been known to get sugar through ant regurgitation [27,28,29]. The consumed sugar can diffuse as trehalose in hemolymph and spread through tissues to reach muscles needed for flight [23]. Energy derived from these sugars also impacts how persistent females are at obtaining blood [13,30] and helps to sustain them in the absence of blood meals [22].

Sugar-feeding is known to be affected by weather (e.g., temperature, humidity), season, and locality (e.g., tropical vs. temperate climate) [13] and is thus likely to be affected by abiotic factors such as temperature. The effect of temperature on blood-feeding is complex as the temperature of the food source, environment, and convection currents from the food source, combined with the environmental temperature, likely affect feeding due to their general effects on the mosquito’s activity [31]. The proposed thermal optimum for biting and blood-feeding has been generally stated to be between 26 and 35 °C and is humidity-dependent [31]. However, the effects of temperature and optimum range for sugar-feeding as well as how temperature possibly impact plant cues (e.g., olfactory), detection, and integration in mosquitoes have yet to be investigated.

Understanding the effects of temperature on sugar-feeding as the global surface temperature is projected to change is particularly important as the Toxic Sugar Bait technique (TSBs) is emerging as a control strategy to target nectar-seeking mosquitoes [32]. The use of TSBs is gaining attention as insecticide resistance rises among mosquito populations [33,34,35]. Moreover, the World Health Organization has urged vector control programs to develop novel strategies for integrated mosquito management (IMM) that are cost-effective, sustainable, and environmentally friendly [36,37]. Fiorenzano et al. [36] recently highlighted that sugar-baiting has been effective in controlling multiple mosquito species including major disease vectors such as *Ae. aegypti* [38], *Culex pipiens* [38], *Ae. albopictus* [39,40,41,42], *Anopheles gambiae* [34,43,44], and *Culex quinquefasciatus* [38,41,44,45], with low impacts on non-target arthropods. A recent study confirmed that sugar feeding is a common behavior of *Ae. aegypti* females in urban areas and suggested that TSBs on plants could be a potentially effective control strategy [46]. Given both the projected expansion of *Ae. aegypti* due to climate change and the potential use of TSB as a control strategy, understanding how temperature affects sugar-feeding in *Ae. aegypti* is a knowledge gap that needs to be filled.

## 2. Materials and Methods

### 2.1. Insects

The strain of *Ae. aegypti* mosquitoes used was Rockefeller (MR-734, MR4, AATCC^®^, Manassas, VA, USA). Larvae were reared in 26 × 35 × 4 cm covered trays that were filled with deionized water with about 200 larvae per tray. The trays were kept in a climatic chamber at 26 °C ± 0.5 °C and 60 ± 10% humidity under light:dark cycles of 12 h:12 h. The diet of the larvae consisted of Hikari Tropic First Bites (Petco, San Diego, CA, USA). For the experiment, around 120 pupae were placed into mosquito breeding containers (BioQuip, Rancho Dominguez, CA, USA—1425, 1425DG) on the day of pupation and until emergence. No sugar was provided to the recently emerged adults before the experiments were conducted.

### 2.2. Actometer Experiments

**Figure 1:**
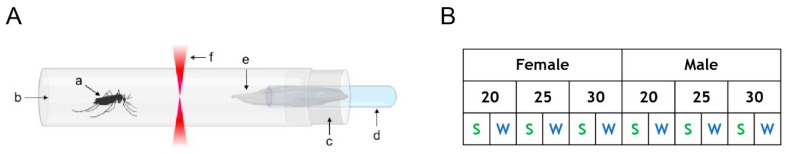
(**A**) Schematic of the setup for monitoring the mosquito activity. A mosquito (a) is placed in a glass tube (b) closed by a cotton plug (c) pierced in its center to fit a cut transfer pipette (d) that is filled with either a 10% sucrose solution or DI water. A cotton mesh (e) is inserted in the transfer pipette and provides access to the solution and maintain a high RH (Relative Humidity) in the tube for the entire experiment. The actometer can hold 32 tubes. (**B**) Table summarizing the twelve different conditions tested. Numbers indicate temperature in °C. S: sugar, W: water.

***Actometer setup***. Mosquito activity was measured using an actometer (Model LAM25, TriKinetics Inc, Waltham, MA, USA) (Figure 1A). Cotton plugs (Genesee Scientific, Morrisville, NC, USA, Cat. 49-102) were cut in half and pierced to fit a plastic transfer pipette (Fisherbrand. Waltham, MA, USA, Cat. 13-711-7M) through. The plastic transfer pipette was then filled with either 10% sucrose (Sigma Aldrich, CAS #57-50-1) solution or DI water depending on which variable was being tested. The water group also serves as a control for a possible effect of humidity on mosquito behavior and thus allowed us to decouple the effect of access to a source of food from the impact of humidity on the general activity. A cotton ball was then rolled up and placed into the pipette bulb so that half of it was in the liquid being tested, and the other half was left outside so the mosquito could have access to it. This technique allowed for the cotton to stay humid for the whole experiment (i.e., 7 days). Then, 1-day-old unfed mosquitoes were collected (32 males or 32 females). After being stored at 4 °C for approximately 5 min, the mosquito container was placed on ice to further prevent the mobility of the mosquitoes. One mosquito was placed in each glass tube and the cotton plug and pipette bulb apparatus were placed to prevent the mosquito from escaping. The tubes were then placed in the actometer which was then placed in the climatic chamber at either 20, 25, or 30 degrees Celsius (°C) (Figure 1B). The relative humidity in the tubes was 80 ± 10%. Each of the twelve conditions was conducted in duplicates (n = 64 mosquitoes per group). The mosquito activity (i.e., the number of beam crossing per 10 min intervals) was recorded for 7 consecutive days using the DAMSystem3 Software (Trikinetics, Waltham, MA, USA). After seven days, the mosquitoes were collected by briefly placing the tubes on ice to anesthetize the individuals and placed in 1.7 microliter tubes for storage at −70 °C until the total carbohydrate content assays were conducted.

***Data analysis***. The activity of the 64 mosquitoes for each condition was analyzed through William’s mean, which is used to support datasets with zero values [47]. Insects that died during the course of the 7-day experiments were recorded and their activity was analyzed and included in the analysis until the last movement was detected by the actometer. Lighting change was accounted for as well through the exclusion and interpolation of the arithmetic mean right before and after the lights were turned off. This helped to avoid bias associated with the increase in activity when the lights were turned off [48,49]. The activity of the different groups was compared time point by time point using a pairwise Student’s *t*-test; *p*-values were adjusted for multiple comparisons with the Bonferroni method using R [50]. Normality was assessed through a Shapiro–Wilk test.

**Total carbohydrates content assays**

**Figure 2:**
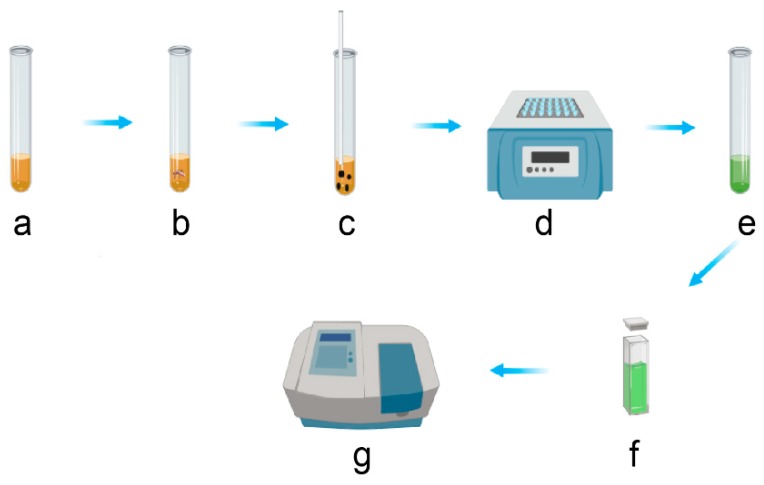
Schematic of the different steps used for the total carbohydrate contents analysis. (**a**) 5 mL of cold anthrone reagent is placed in a test tube; (**b**) A single mosquito is added to the anthrone and (**c**) crushed. (**d**) The mixture is heated at 92 °C for 17 min and (**e**) cooled down before being (**f**) transferred to a cuvette. (**g**) The absorbance is read using a spectrophotometer and later converted to sugar content.

Total carbohydrates contents were measured using the method described by van Handel [51] (Figure 2). Briefly, anthrone (Sigma-Aldrich CAS #90-44-8) reagent was prepared by combining 150 mL water in a 1-L Erlenmeyer flask on ice with 380 mL sulfuric acid (Fisher CAS #7664-93-9), in which 750 mg of anthrone was then dissolved. Each mosquito analyzed was placed in a culture glass tube (Sigma-Aldrich C1048-72EA) that was filled to a 5 mL mark with anthrone reagent and then crushed with a glass rod. The sample was heated for 17 min at 92 °C in a dry bath, then cooled before being vortexed for 15–20 s. A sample without a mosquito was prepared additionally as a blank for the spectrophotometer (Perkin Elmer Lambda 20 UV/Visible Spectrophotometer). The optical density (OD) of each sample was then determined at 625 nm. For samples with an OD_625_ above one, 200 μL of sample was diluted with 800 μL anthrone reagent that had been heated as above, giving a dilution factor of 5. The carbohydrate content was quantified using the OD values and a calibration line that had been created by performing the above procedure with samples containing 25, 50, 100, 150, and 200 μg of glucose solution. This assay was performed with both male and female *Ae. aegypti* mosquitoes that were 7 days old and alive at the end of the actometer experiments.

***Data analysis***. Normality was assessed through a Shapiro–Wilk test. A two-way ANOVA followed by a Tukey HSD post hoc test was used to assess differences between groups using R.

## 3. Results

### 3.1. Actometer Experiments

**Figure 3:**
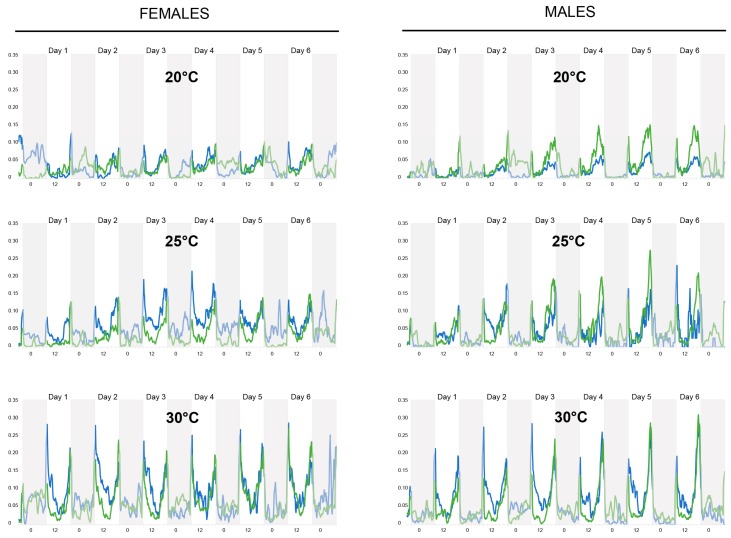
Activity results for females (**left**) and males (**right**) at the three different temperatures tested (20, 25, and 30 °C). Blue lines exhibit results for the water-fed mosquitoes while green lines exhibit results for the sucrose-fed mosquitoes. Each line is the average activity (Williams’ mean) (y-axis) of 64 mosquitoes. Grey vertical bars indicate nighttime and white vertical bars indicate daytime. The x-axis represents time: 0 = midnight, 12 = noon.

*Activity*. In both females and males, the activity increased with temperature (pairwise *t*-test with Bonferroni correction, for all comparisons: *p* < 0.001) (Figure 3). In females, the water-fed groups were significantly more active than the sugar-fed group at all three tested temperatures (*t*-test, for all comparisons: *p* < 0.001). However, in males, access to a sugar source increased activity at 20 and 25 °C but not at 30 °C. Overall, water-fed females tended to have a higher flying activity than water-fed males (*t*-tests, for all comparisons: *p* < 0.001). However, when provided with sucrose, males were more active at 20 and 25 °C (*t*-tests, for all comparisons: *p* < 0.001) compared to females, but not at 30 °C (*t*-test, *p* = 1). Interestingly, we noticed some nocturnal activity in both females and males under all conditions.

**Figure 4:**
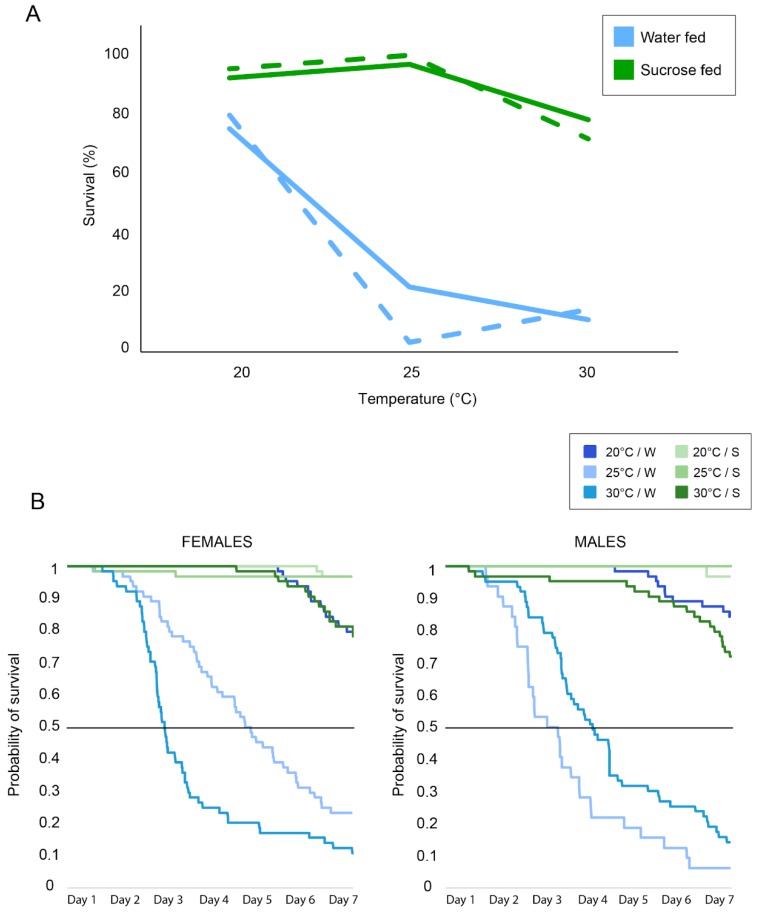
(**A**) Survival (in percentages) after the seven-day actometer experiments at different temperature regimes for females (plain lines) and males (dashed lines) when fed with sucrose (in green) or maintained on water (in blue). (**B**) Raw (Kaplan–Meier) survival data throughout the course of the seven-day experiments for the different conditions tested in females (left) and males (right). The black line indicates 50% of mosquito mortality.

*Survival*. In both females and males, sucrose increases the survival of mosquitoes compared to the water groups (Figure 4A). However, females and males did not show difference in their survival. We then performed Log-rank tests comparing survival curves [52] built on our Kaplan–Meier estimates of the survival probability [53] (Figure 4B). We noted a significant difference in survival between the different treatments for both females and males (Log-rank test, *p* < 0.001).

In females, access to sugar had a significant positive impact on survival (Log-rank tests, for all comparisons *p* < 0.001). A strong effect of temperature on survival was noted in the water groups (Log-rank tests, for all comparisons *p* < 0.001). However, access to sucrose minimized the effect of temperature between groups maintained at 20 °C and 25 °C (Log-rank test *p* = 1) but not for the other groups. Both female groups with access to sugar at 20 °C and 25 °C had significantly higher survival rates (92.19% and 96.88%, respectively) than the sugar-fed female group at 30 °C (78.13%) (Log-rank test, *p* = 0.001 and *p* = 0.002, respectively). In males, no significant difference between sucrose and water-fed groups was found at 20 °C (Log-rank test *p* = 0.07), while at 25 °C and 30 °C, sugar access significantly increased the mosquito survival (Log-rank test, *p* < 0.001). Temperature had a significant impact on survival for both the sucrose groups and water groups (Log-rank test, for all comparisons *p* < 0.001). For water-fed males, the 20 °C group had the highest survival rate (79.69%), followed by the 30 °C group (14.06%) and the 25 °C group (3.13%). For the sugar-fed males, the 25 °C group had the highest survival rate (100.00%), followed by the 20 °C group (79.69%) and the 30 °C group (71.88%).

The average number of days lived, including mosquitoes that survived during the whole experiment and ones that did not, was higher for females which had access to sugar (20 °C: 6.51 days; 25 °C: 6.38 days; 30 °C: 6.27 days) compared to the water-fed groups (20 °C: 6.35 days; 25 °C: 4.2 days; 30 °C: 2.88 days) (*t*-test, for all comparisons, *p* < 0.001). This was also observed for males (sucrose groups: 20 °C: 6.5 days; 25 °C: 6.06 days; 30 °C: 6.54 days; water groups: 20 °C: 6.24 days; 25 °C: 3.67 days; 30 °C: 2.78 days) (*t*-test, for all comparisons, *p* < 0.001).

### 3.2. Total Carbohydrates Content Assays

Upon generation of total carbohydrate contents using van Handel’s method (Figure 2), any statistical variance between differing experimental groups was sought out using a *two-way* ANOVA, followed by a Tukey HSD post hoc test. A summary of how each variable or combination thereof plays a role in affecting the overall carbohydrate content of *Ae. aegypti* is displayed in Table 1. Each individual variable (i.e., temperature, food source, and sex) had a statistically significant effect on carbohydrate concentrations (for all comparisons, *p* < 0.001). In addition, these three variables in conjunction with one another were shown to also affect carbohydrate levels (*p* = 0.004).

**Figure 5:**
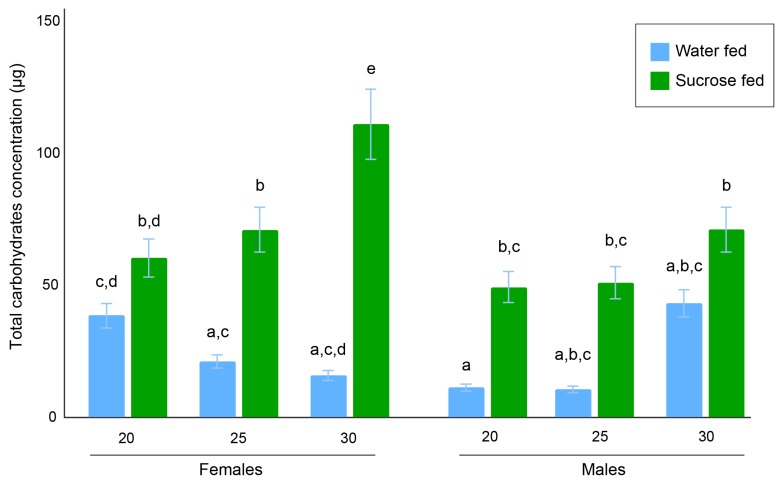
Bar plot of the total carbohydrate contents in females and males. Bars indicate the standard error of the mean. Letters above bars denote statistical differences between groups. (Females—20 °C WF: n = 48, SF: n = 58; 25 °C WF: n = 14, SF: n = 61; 30 °C WF n = 7, SF n = 50; Males—20 °C WF n = 50, SF: n = 60; 25 °C WF n = 2, SF n = 64; 30 °C WF n = 9, SF n = 25). WF = water-fed; SF = sucrose-fed.

Following the ANOVA, we performed a Tukey HSD post hoc test to determine the statistical differences between each of the 12 experimental groups (Figure 5).

In females, the 30 °C sugar-fed group had the highest relative sugar concentration (108.01 ± 9.33 µg) and was statistically different from the 20 °C sugar-fed group (60.10 ± 4.98 µg) and 25 °C sugar-fed group (70.85 ± 6.77 µg) (*p* < 0.01). However, no significant difference was detected between the 20 °C and 25 °C sugar-fed groups (*p* = 0.94). In addition, the 25 °C sugar-fed group was significantly different from the three water-fed female groups (*p* < 0.01 for 20 °C and 25 °C and *p* = 0.02 for 30 °C), for which sugar concentrations were 38.59 ± 6.42 µg, 20.9 ± 5.47 µg, 15.61 ± 2.79 µg, respectively.

In males, the 20 °C and 25 °C sugar-fed groups had sugar contents of 49.12 ± 2.86 µg and 50.77 ± 3.62 µg, respectively, and no significant difference was found (*p* = 1). The 30 °C sugar-fed male group had a higher sugar content (71.10 ± 6.33 µg) but was not significantly different than the 20 °C or 25 °C groups (*p* = 0.16 and *p* = 0.24, respectively). Each of the sugar-fed groups had a significantly (*p* < 0.01) higher sugar content than the 20 °C water-fed group (11.02 ± 0.66 µg). Statistical differences were not observed between the sugar-fed and water-fed groups when compared with the 25 °C (10.36 ± 3.79 µg) and 30 °C (42.90 ± 13.67 µg). This may be due to the small sample sizes due to high mortality in these two groups (n = 2 for the 25 °C water-fed males and n = 9 for the 30 °C males).

When comparing females and males, we found that the 20 °C water-fed males and females were significantly different (*p* = 0.0281). The 25 °C water-fed female and male groups were not significantly different (*p* = 0.15), although this may be related to the small sample size of the 25 °C male water-fed group (n = 2). The 30 °C water-fed female and male groups were also not significantly different, but this may also be due to small sample sizes. The sugar content of the 20 °C sugar-fed females was not significantly different than the 20 °C sugar-fed males (*p* = 0.96), and the 25 °C sugar-fed females’ sugar content was also not significantly different than the 25 °C sugar-fed males’ sugar content (*p* = 0.15). A significant difference (*p* < 0.01) was found between the 30 °C sugar-fed females and 30 °C sugar-fed males.

## 4. Discussion

The daily patterns of activity and survival results from the actometer experiments provide essential insights into how females and males are affected by temperature and how this effect is mediated by access to sugar. In the present study, we show that sugar deprivation increases activity in females at all tested temperatures while sugar deprivation only increases males’ activity at 30 °C and decreases it at 20 °C and 25 °C. It is worth mentioning that the activity results for the females at 30 °C and for the males at 25 °C and 30 °C may be influenced by the low number of surviving mosquitoes in those groups. Overall, males had a higher level of activity when they had access to sugar. This can be explained by the fact that males rely entirely on sugar feeding to sustain their metabolism and have lower energetic reserves compared to females [13,54]. Males also take smaller sugar meals and are required to seek for nectar more often than females, which need carbohydrates, but can also rely on blood as a source of water and nutrients [13,55]. In the absence of sugar, the females may have increased activity because of their higher nutrient pools carried over from the larval stage that can be used as a source of energy for flight [54].

Our results show that access to sugar improved survival for both females and males across all three tested temperatures. Interestingly, most females (~75%) and males (~80%), when maintained at 20 °C, were able to survive the whole experiment (7 days) without access to sugar, thus highlighting their resilience and tolerance to an environment with limited resources. This indicates that under cool temperatures, mosquitoes can easily survive without access to nectar by decreasing their general activity and use stored energy reserves while waiting for more favorable conditions to return. Higher temperature had an overall negative impact on survival rates, although access to sugar minimized this effect. These results agree with Costa et al. [12], who found that survival rates decreased as temperature increased from 25 °C to 30 °C and 35 °C. The seemingly optimum temperature range around 25 °C indicates that in the face of climate change, regions with temperatures nearing closer to 30 °C may experience declining *Ae. aegypti* populations and associated diseases, while areas with temperature averages rising to around 25 °C may see increases in *Ae. aegypti* populations and disease.

Our actometer data clearly show the two peaks of activity (i.e., at dawn and dusk) that have been previously reported in this species [31]. Interestingly, we also show that the mosquitoes (both females and males) were active at night. *Ae. aegypti* has been classically considered a day-active species, but our data indicate that nocturnal activity also occurs. This is consistent with previous reports of nectar-feeding activity during the night or at dusk/dawn in several mosquito species, both diurnal and nocturnal [23,56,57,58]. Indeed, sugar-feeding in wild mosquitoes has been observed to have diel periodicities, suggesting that an endogenous rhythm underlies this behavior, although it has been found that sugar-feeding is also dependent on the time in relationship to the time of sunrise and sunset [23,59].

The total carbohydrates assay indicates that the sugar-fed mosquitoes consumed the most at 30 °C, as both females and males had the highest sugar content among all groups. This agrees with previous findings that insect sugar consumption increases with temperatures between 20 °C and 30 °C [60]. This also fits with the actometer results that show that both sexes had the highest activity at higher temperatures because higher activity would likely indicate more frequent visitation of the sugar source, and the higher activity would be enabled by the higher energy consumption. This positive relationship may be caused by the increase in metabolism that occurs at elevated temperatures [61,62,63,64] and triggers an increase in the mosquito activity which enables them to eat enough to upkeep with their metabolism. This would agree with a recent observation by Klepsatel et al. [63] who reported a positive correlation between metabolism and temperature, as well as food consumption and temperature in *Drosophila*. Conversely, lower temperatures led to lower activity, likely because the metabolic rates were lower, thus the mosquitoes did not need to eat as much to meet their metabolic energy demands.

Toxic sugar baits have emerged as an important tool for controlling mosquito populations and may prove to be particularly useful for *Ae. aegypti* and other disease vectors in light of this positive relationship between sugar-feeding and temperature and the projected global warming temperature increases. Rising temperatures will likely raise metabolic rates and affect levels of water and thus cause higher sugar consumption. Furthermore, understanding how much sugar is consumed at different temperatures, which, as shown here, influences flying activity, could be a consideration when determining dose concentrations of the sugar baits. Determining a precise toxin concentration for the TSBs based on fluctuating weekly environmental temperatures may prove to make them even more cost-effective and powerful. TSBs during the warmest months could be more cost-effective and use lower doses because the temperature should cause the mosquitoes to eat more, thus a lower toxin concentration would be needed. Additionally, using projected global temperature changes to determine what geographic regions will have optimum sugar-feeding conditions can inform on what areas may benefit the most from TSB use.

This study sheds light on the combinatorial effects of constant temperature, sugar availability, and activity in an important disease vector mosquito species. The next step is to conduct assays with fluctuating temperatures (i.e., cooler nights and warmer days) to reflect current natural settings and predicted ones to observe the potential impact on the mosquito activity in the presence or absence of a sugar source. This will lead us to have a better understanding of how global warming might affect general mosquito activity and survival, and consequently its effect on their global distribution and population dynamics. This is critical to predict so that regions most likely to have high population densities are equipped with supplies to control and mitigate their populations and accompanying diseases. As sugar sources are variable throughout the year (at least in temperate regions) (reviewed by 13), it also appears essential to get a better knowledge of the potential sources of nectar that mosquitoes feed on in the field. As climate evolves, certain plants might have a longer/shorter blooming seasons which might in return affect mosquito population dynamics. Additionally, as the effects of temperature on *Aedes* survivability and oviposition have been found to be humidity level dependent [12,65], future assays could investigate the impact of humidity on sugar-feeding as well. Understanding this factor’s effect will enable a more global understanding of climate change’s potential effect on mosquito population distribution and season duration as global warming is projected to change global humidity levels [1]. Future related work could also investigate the effects of dehydration on sugar-feeding as drought frequency is predicted to increase with global warming [66], and dehydrated mosquitoes have been shown to have heightened activity levels and blood-feeding behavior [67]. Finally, we conducted this work using a well-established line of *Ae. aegypti* that has been maintained for many generations under laboratory conditions. It would thus be interesting to compare the present results with data from field-caught mosquitoes. Overall, determining how changing temperatures and humidity will affect *Ae. aegypti* behavior (including sugar-feeding) is crucial for understanding how population distribution and dynamics will be affected.

## 5. Conclusions

Combining actometer experiments and calorimetric assays, we studied the impact of temperature and access to sugar on *Ae. aegypti* mosquitoes’ activity and survival. We show that access to sugar increases survival in both females and males and that activity was also correlated with access to sugars. The temperature had a strong effect on the general activity in both sexes and on carbohydrates consumption and storage. This study is the first to assess the combined effects of temperature and access to a sugar source on females and males *Ae. aegypti* mosquitoes’ daily activity patterns. It is of particular importance in the context of climate change and the emergence of new control tools such as the Toxic Sugar Baits. This study constitutes the first step of investigation on the extent to which temperature, and in particular future climate, might affect mosquito distribution and how access to sugar might contribute to their overall fitness.

## Figures and Tables

**Table 1:** Summary table for the two-way ANOVA analysis of the impact of the access to sugar, sex, and temperature on the total carbohydrate concentrations in *Aedes aegypti* mosquitoes.

Factors	Df	Sum Sq	Mean Sq	F Value	Pr(>F)	Significance
Temperature	2	121,442	60,721	37.876	6.09 × 10^−16^	***
Food source	1	110,499	110,499	68.926	1.18 × 10^−15^	***
Sex	1	49,724	49,724	31.016	4.38 × 10^−8^	***
Temperature:Food source	2	10,465	5232	3.264	0.03914	*
Temperature:Sex	2	1628	814	0.508	0.6022	
Food source:Sex	1	6	6	0.004	0.9522	
Temperature:Food source:Sex	2	17,728	8864	5.529	0.00424	**
Residuals	455	729,438	1603			
